# Investigation of the feasibility of elective irradiation to neck level Ib using intensity-modulated radiotherapy for patients with nasopharyngeal carcinoma: a retrospective analysis

**DOI:** 10.1186/s12885-015-1669-z

**Published:** 2015-10-15

**Authors:** Fan Zhang, Yi-Kan Cheng, Wen-Fei Li, Rui Guo, Lei Chen, Ying Sun, Yan-Ping Mao, Guan-Qun Zhou, Xu Liu, Li-Zhi Liu, Ai-Hua Lin, Ling-Long Tang, Jun Ma

**Affiliations:** 1 Department of Radiation Oncology, State Key Laboratory of Oncology in South China, Collaborative Innovation Center for Cancer Medicine, Sun Yat-sen University Cancer Center, No. 651 Dongfeng Road East, Guangzhou, 510060 People’s Republic of China; 2 Department of Radiation Oncology, The Sixth Affiliated Hospital of Sun Yat-sen University, Guangzhou, 510655 People’s Republic of China; 3 State Key Laboratory of Oncology in South China, Collaborative Innovation Center for Cancer Medicine, Imaging Diagnosis and Interventional Center, Sun Yat-sen University Cancer Center, Guangzhou, 510060 People’s Republic of China; 4 Department of Medical Statistics and Epidemiology, School of Public Health, Sun Yat-sen University, Guangzhou, 510080 People’s Republic of China

**Keywords:** Nasopharyngeal neoplasms, Intensity-modulated radiotherapy, Elective neck irradiation, Level Ib

## Abstract

**Background:**

To assess the feasibility of elective neck irradiation to level Ib in nasopharyngeal carcinoma (NPC) using intensity-modulated radiation therapy (IMRT).

**Methods:**

We retrospectively analyzed 1438 patients with newly-diagnosed, non-metastatic and biopsy-proven NPC treated with IMRT.

**Results:**

Greatest dimension of level IIa LNs (DLN-IIa) ≥ 20 mm and/or level IIa LNs with extracapsular spread (ES), oropharynx involvement and positive bilateral cervical lymph nodes (CLNs) were independently significantly associated with metastasis to level Ib LN at diagnosis. No recurrence at level Ib was observed in the 904 patients without these characteristics (median follow-up, 38.7 months; range, 1.3–57.8 months), these patients were classified as low risk. Level Ib irradiation was not an independent risk factor for locoregional failure-free survival, distant failure-free survival, failure-free survival or overall survival in low risk patients. The frequency of grade ≥ 2 subjective xerostomia at 12 months after radiotherapy was not significantly different between low risk patients who received level Ib-sparing, unilateral level Ib-covering or bilateral level Ib-covering IMRT.

**Conclusion:**

Level Ib-sparing IMRT should be safe and feasible for patients without a DLN-IIa ≥ 20 mm and/or level IIa LNs with ES, positive bilateral CLNs or oropharynx involvement at diagnosis. Further investigations based on specific criteria for dose constraints for the submandibular glands are warranted to confirm the benefit of elective level Ib irradiation.

## Background

Nasopharyngeal carcinoma (NPC) is one of the most common head and neck malignancies in Southeast Asia. Radiotherapy is the mainstay treatment modality for NPC. Intensity-modulated radiation therapy (IMRT) has gradually replaced two-dimensional radiation therapy (2D-RT) as it offers improved target conformity, arousing a need for evidence of how to feasibly reduce specific radiation fields and provide better protection of adjacent organs at risk (OARs) without jeopardizing disease control [1, 2]. Xerostomia is the most common side effect of radiotherapy in NPC. Most stimulated saliva is secreted by the parotid glands (PGs), while the submandibular glands (SMGs) produce most of the unstimulated saliva and mucins, which may influence the degree of a dry mouth sensation [3]. Preliminary data demonstrated that IMRT can spare the PGs to aid recovery of secretion [4, 5] and confirmed protection of the SMGs can speed up the recovery of salivary flow and reduce xerostomia [6–10]. Therefore preservation of SMG function during IMRT is crucial to reduce xerostomia.

The SMGs are located in neck node level Ib. Previous studies revealed that level Ib is not a regular region of direct drainage [11, 12] and skip metastasis in the cervical nodes is extremely infrequent in NPC [11, 13, 14]. The incidence of level Ib lymph node (LN) involvement is low in NPC (range 2–4 %) [11, 13–15]. Therefore, it may be safe to selectively omit level Ib irradiation in certain groups of patients with NPC treated using IMRT. However, there is no consensus on this issue. Some studies routinely irradiate level Ib [1, 16–18], which exposes the SMGs to radiation; whereas others selectively spare level Ib with different criteria [11, 19–21]. Data on elective neck irradiation to level Ib in patients with NPC treated with IMRT is scarce. Chen and colleagues [22] reported that regional LN recurrence alone is rare in patients with negative level Ib LNs after level Ib-sparing IMRT; however, suitable criteria for elective irradiation of neck level Ib need to be re-evaluated due to the small sample size investigated.

To provide the optimal balance between preservation of the SMGs and regional control, it necessary to investigate which cohorts of patients can be spared level Ib irradiation. Therefore, we conducted a retrospective study to assess the feasibility of elective level Ib irradiation in a large cohort of patients with NPC treated with IMRT.

## Methods

### Patients

Approval for retrospective analysis of the patient data was obtained from the ethics committee of Sun Yat-sen University Cancer Center. Informed consent was obtained from each patient for their consent to have their information used in research without affecting their treatment option or violating their privacy. Selection criteria were: (1) patients with newly-diagnosed, histologically-confirmed NPC; (2) with no evidence of distant metastasis (M0); (3) who completed the planned course of radical IMRT; (4) and for whom full treatment plan data was available, including the isodose distribution and dose-volume histogram (DVH). Exclusion criteria included: (1) prior or other current malignancy; (2) prior RT, chemotherapy or surgery (except for diagnostic procedures) to the primary tumor or nodes. Between November 2009 and December 2012, 1811 consecutive patients with newly-diagnosed, non-metastatic, biopsy-proven NPC were treated with IMRT at our center. All patients underwent a pretreatment evaluation, including complete history, physical and neurologic examinations, hematology and biochemistry profiles, MRI scans of the nasopharynx and neck, chest radiography, abdominal sonography and single photon emission computed tomography (SPECT). Furthermore, 29.2 % (528/1811) underwent positron emission tomography (PET)-CT. Medical records and imaging studies were analyzed retrospectively. All patients were restaged according to the 7th edition of the American Joint Committee on Cancer (AJCC) staging system for NPC. Of these, 373 (20.5 %) patients were eliminated from the study, as their treatment plans were incomplete due to data loss (damage to hard disk) and unavailable for further analyses. The resulting 1438 patients were included in this study.

### Image assessment

All MRI materials and clinical records were retrospectively reviewed to minimize heterogeneity in restaging. All scans were separately evaluated by two radiologists specializing in head-and-neck cancer (Ying Sun and Li-Zhi Liu,); all disagreements were resolved by consensus. Nodal size data (for example, the maximal axial diameter and minimal axial diameter), necrosis and extracapsular spread (ES) for positive LNs were documented. The diagnostic criteria for retropharyngeal lymph node (RLN) and cervical lymph node (CLN) metastases included (1) any visible LN in the median RLNs, a shortest axial dimension ≥ 5 mm in the lateral RLNs, ≥ 11 mm for the jugulodigastric region and ≥ 10 mm in other cervical regions, or a group of three LNs that were borderline in size; or (2) LNs of any size in the presence of necrosis or ES [23, 24]. The criteria for the diagnosis of central necrosis on MRI were a focal area of high signal intensity on T2-weighted images or a focal area of low signal intensity on T1-weighted images with or without a surrounding rim of enhancement; the criteria for extracapsular spread were the presence of indistinct LN margins, irregular LN capsular enhancement, or infiltration into the adjacent fat or muscle [24]. Lymph node locations were based on the International Consensus Guidelines for neck level delineation [12].

### Radiotherapy

All patients received IMRT. All patients were immobilized in the supine position with a thermoplastic mask. After administration of intravenous contrast material, 3 mm CT slices were acquired from the head to the level 2 cm below the sternoclavicular joint. Target volumes were defined in accordance with International Commission on Radiation Units and Measurements reports 50 and 62. All target volumes were delineated slice-by-slice on the treatment planning computed tomography scan as follows:

(i) GTV (Gross Tumor Volume): determined from MRI, clinical information, and endoscopic findings. Gross disease at the primary site together with enlarged RLNs was designated as the GTVnx and clinically-involved gross LNs were designated as the GTVnd.
(ii) CTV (clinical target volumes): were individually delineated on the basis of the tumor invasion pattern [14]. The first clinical tumor volume (CTV-1) was defined as the GTVnx plus a 5–10-mm margin for the high-risk regions of microscopic extension encompassing the entire nasopharynx. The second CTV (CTV-2) was defined by adding a 5–10 mm margin to CTV-1 for low-risk regions of microscopic extension (this margin could be reduced where CTV-2 was in close proximity to critical structures) and included the entire nasopharynx, anterior half to two-thirds of the clivus (or entire clivus, if involved), skull base (bilateral foramen ovale and rotundum), pterygoid fossae, parapharyngeal space, inferior sphenoid sinus (in T3-T4 disease, the entire sphenoid sinus), posterior quarter to third of the nasal cavity, maxillary sinuses (to ensure pterygopalatine fossae coverage), the levels of the LNs located, and the elective neck area. Neck levels were contoured according to the International Consensus Guidelines for the CT-based delineation of neck levels published in 2003 [12]. The elective neck area included either partial neck irradiation of levels II, III and VA or whole neck irradiation of level II-V. This decision was made by the individual doctors for each case. In respect of neck irradiation of neck node level Ib for the 1398 patients without metastasis to the level Ib LNs at diagnosis, 31.7 % (443/1398) patients received irradiation of level Ib (level Ib-covering IMRT, including unilateral level Ib in 16.5 % [231/1398] and bilateral level Ib in 15.2 % [212/1398]); the remainder (68.3 %, 955/1398) received level Ib-sparing IMRT.
(iii) The OARs: included the brainstem, spinal cord, temporal lobe, optic nerves, optic chiasm, lens, eyes, parotid glands, mandible, temporomandibular joints, middle-ears and larynx.

**Fig. 1:**
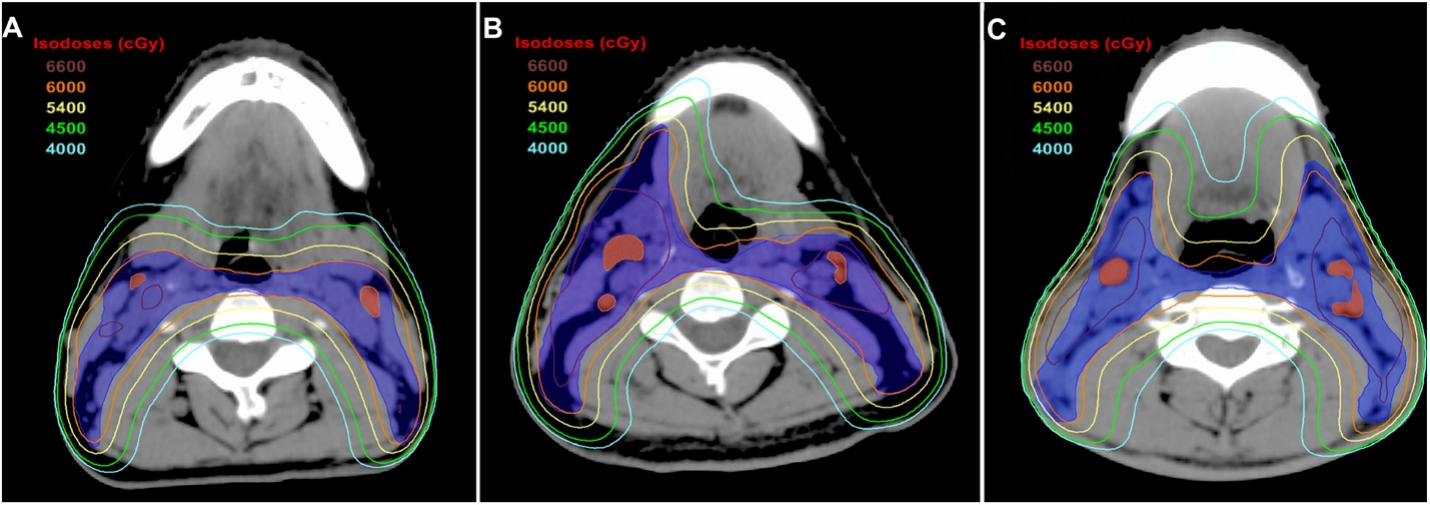
Isodose distributions for the submandibular glands. The 40 Gy and higher isodose distributions for the posterior part of the SMGs and anterior part of the SMGs in patients with NPC who received level Ib-sparing IMRT (**a**), unilateral level Ib-covering IMRT (**b**), and bilateral level Ib-covering IMRT (**c**). *CTV-2, blue shadow; GTV-LN, red shadow; 66 Gy isodose, brown line; 60 Gy isodose, orange line; 54 Gy isodose, yellow line; 45 Gy isodose, green line; 40 Gy isodose, blue line*

The prescribed radiation doses were: a median total dose of 68 Gy (range, 66–72 Gy) in 30–33 fractions to the planning target volume (PTV) of GTV-P, 64 Gy (range, 64–70 Gy) to the PTV of the nodal gross tumor volume (GTV-N), 60 Gy (range, 60–63 Gy) to the PTV of CTV-1, and 54 Gy (range 54–56 Gy) to the PTV of CTV-2 (low-risk regions and neck nodal regions). The constraints for the OARs were as per the Radiation Therapy Oncology Group (RTOG) guidelines as reported in a previous study (Brain stem: Dmax ≤ 54 Gy, Brain stem PRV: D1% ≤ 60 Gy; Spinal cord: Dmax ≤ 45 Gy, Spinal cord PRV: D1% ≤ 50 Gy; Optic nerves, Chiasm: Dmax ≤ 54 Gy; Parotid glands: Dmean ≤ 26 Gy, V30 ≤ 50 %) [25]. However, as delineation of the SMGs was described in the protocol of our centre, there was no dose constraint for the SMGs. Fig. 1 shows the ≥ 40 Gy isodose distributions for the posterior and anterior regions of the SMGs. All patients were treated with one fraction daily 5 days per week. Intracavitary after-loading treatment with iridium-192 was used to address local persistence at 3–4 weeks after external RT at 15 to 20 Gy in three to five fractions every 2 days.

### Chemotherapy

During the study period, institutional guidelines recommended no chemotherapy in stage I–IIA, concurrent chemoradiotherapy in stage IIB, and concurrent chemoradiotherapy with or without induction/adjuvant chemotherapy for stage III–IVA-B, as defined by the 7th edition of the UICC/AJCC Staging System. Overall, 203/1438 patients (14.1 %) were treated with RT only, and 1235/1438 patients (85.9 %) received induction, concurrent, or adjuvant chemotherapy (concurrent alone, 35.5 % [511/1235]; induction-concurrent, 37.4 % [538/1235]; concurrent-adjuvant, 1.1 % [14/1235]; 0.9 % induction-concurrent-adjuvant [13/1235]; 10.9 % induction alone, [156/1235]). In total, 93.0 % (996/1071) of patients with stage III–IV disease received chemotherapy. Deviations from institutional guidelines were due to organ dysfunction (suggesting intolerance to chemotherapy) or patient’s refusal.

### Follow-up and xerostomia assessment

Follow-up was measured from first day of treatment to day of last examination or death. During the first two years, patients were evaluated every three months, and every six months thereafter for 3 year or until death. Generally, follow-up included physical and neurologic examinations, chest radiography, abdominal sonography, single photon emission CT whole body bone scan, and head and neck MRI. All local recurrences were diagnosed by soft-tissue swelling in fiberoptic endoscopy or MRI of the nasopharynx and confirmed by biopsy, except for recurrence at the skull base which was confirmed by progressive bone erosion on MRI. Regional recurrences were diagnosed by clinical examination or neck MRI and confirmed by biopsy. Distant metastases were diagnosed by clinical symptoms, physical examinations, and imaging methods including chest radiography, bone scan, MRI, CT and abdominal sonography. Xerostomia related to radiation therapy was graded at approximately 12 months after radiotherapy according to the Radiation Morbidity Scoring Criteria of the RTOG.

### Statistical analysis

All analyses were conducted using Statistical Package for the Social Sciences 19.0 (SPSS; Chicago, IL, USA). All events were measured from the first day of treatment. The following endpoints (interval to the first defining event) were evaluated: locoregional failure-free survival (LR-FFS), distant failure-free survival (D-FFS), failure-free survival (FFS) and overall survival (OS). LR-FFS was calculated from the first date of treatment to first locoregional failure; D-FFS, to first remote failure; FFS, to the date of tumor relapse or death from any cause, whichever occurred first; and OS, to last examination or death.

To investigate predictors for neck level Ib metastasis at diagnosis, the Chi-square test (or Fisher’s exact test, if indicated) was employed for univariable analyses to examine associations and a logistic regression model, for multivariable analyses to estimate hazard ratios (HR) and test independent significance by backward elimination of insignificant explanatory variables.

To investigate whether irradiation of level Ib was associated with xerostomia, regional and subsequent distant control, the Chi-square test (or Fisher’s exact test, if indicated) was used to evaluate the baseline clinical characteristics and the degree of xerostomia. Actuarial survival rates were estimated by the Kaplan-Meier method and compared using the log-rank test. Multivariable analyses using the Cox proportional hazards model were used to estimate hazard ratios (HR) and test independent significance by backward elimination of insignificant explanatory variables. Statistical significance was defined as *P* <0.05 based on two-sided tests.

## Results

### Predictors for metastasis to the level Ib lymph nodes at diagnosis

Univariable analysis of 1438 patients revealed that more advanced N disease (for example, greatest dimension of the level IIa LNs [DLN-IIa] ≥ 20 mm or level IIa LNs with ES [*P <*.001]) and orpharynx involvement (*P* = .001) were significantly associated with metastasis to the level Ib LNs at diagnosis (Table 1).

**Table 1:** Univariable analyses of factors related to level IB LNs metastases at diagnosis in 1438 patients

Variable	Metastasis to level Ib LNs at diagnosis, *N* (%)		*P
	(−), *n* = 1398	(+), *n* = 40	
Sex			
Male	1052 (75.3)	33 (82.5)	.294
Female	346 (24.7)	7 (17.5)	
Age			
<50 years	950 (68.0)	23 (57.5)	.163
≥50 years	448 (32.0)	17 (42.5)	
Histologic type			
Keratinizing squamous cell carcinoma	5 (0.4)	0	1.000
Nonkeratinizing carcinoma	1393 (99.6)	40 (100.0)	
T stage			’
T1	247 (17.7)	5 (12.5)	.537
T2	207 (14.8)	6 (15.0)	
T3	679 (48.6)	18 (45.0)	
T4	265 (19.0)	11 (27.5)	
T classification			
T1-3	1133 (81.0)	29 (72.5)	.176
T4	265 (19.0)	11 (27.5)	
Oropharynx involvement			
(−)	1291 (92.3)	31 (77.5)	.001
(+)	107 (7.7)	9 (22.5)	
Nasal cavity involvement			
(−)	918 (65.7)	22 (55.0)	.162
(+)	480 (34.3)	18 (45.0)	
N classification			
N0	235 (16.8)	0	<.001
N1	823 (58.9)	19 (47.5)	
N2	216 (15.5)	13 (32.5)	
N3	124 (8.9)	8 (20.1)	
Positive RLNs			
(−)	387 (27.7)	3 (7.5)	.005
(+)	1011 (72.3)	37 (92.5)	
Positive CLNs			
(−)	570 (40.8)	4 (10.0)	<.001
(+)	828 (59.2)	36 (90.0)	
LN necrosis			
(−)	1054 (75.4)	22 (55.0)	<.001
(+)	344 (24.6)	18 (45.0)	
LNs with ES			
(−)	1051 (75.2)	26 (65.0)	.143
(+)	347 (24.8)	14 (35.0)	
D_LN-IIa_ ≥30 mm or level IIa LNs with ES			
(−)	1247 (89.2)	34 (85.0)	.435
(+)	151 (10.8)	6 (15.0)	
D_LN-IIa_ ≥20 mm or level IIa LNs with ES			
(−)	1113 (79.6)	19 (47.5)	<.001
(+)	285 (20.4)	21 (52.5)	
MAD of LNs ≥30 mm			
(−)	1196 (85.6)	26 (65.0)	<.001
(+)	202 (14.4)	14 (35.0)	
Positive bilateral CLNs			
(−)	1121 (80.2)	20 (50.0)	<.001
(+)	277 (19.8)	20 (50.0)	
Positive CLNs at supraclavicular fossa			
(−)	1318 (94.3)	21 (80.0)	<.001
(+)	80 (5.7)	8 (20.0)	

Abbreviations: *LNs*, lymph nodes; *WHO*, World Health Organization; *RLNs*, retropharyngeal lymph nodes; *CLNs*, cervical lymph nodes; *LNs*, lymph nodes; *DLN-IIa*, greatest dimension of level IIa lymph nodes; *MAD*, maximal axial diameter; *ES*, extra-capsular spread

*P-values were calculated using an unadjusted chi-square test (or Fisher’s exact test, if indicated)

Multivariable analysis to adjust for various risk factors demonstrated a DLN-IIa ≥ 20 mm or level IIa LNs with ES (HR 2.21; 95 % confidence interval [CI] 1.10–4.46; *P* = .026) and oropharynx involvement (HR 2.59; 95 % CI 1.18–5.69; *P* = .018) were independently significantly associated with metastasis to the level Ib LNs at diagnosis, while positive bilateral CLNs (HR 1.95; 95 % CI 0.97–3.92; *P* = .061) had a borderline significant association with metastasis to the level Ib LNs at diagnosis (Table 2). In the 1193 patients with positive LNs in this series, univariable and multivariable analyses confirmed that a DLN-IIa ≥ 20 mm and/or level IIa LNs with ES (HR 2.41; 95 % CI 1.22–4.76; *P* = .011), oropharynx involvement (HR 2.50; 95 % CI 1.13–5.56; *P* = .024) and positive bilateral CLNs (HR 2.11; 95 % CI 1.06–4.20; *P* = .034) were independently significantly associated with metastasis to the level Ib LNs at diagnosis.

**Table 2:** Multivariable analysis of predictors for level IB LNs metastases at diagnosis in 1438 patients

Variable	HR	95 % CI	*P* ^*^
Age, ≧50 years vs. <50 years	1.51	0.78–2.94	.219
T classification, T4 vs. T1-3	1.16	0.53–2.52	.708
Nasal cavity involvement, (+) vs. (−)	1.31	0.65–2.64	.446
Oropharynx involvement, (+) vs. (−)	2.59	1.18–5.69	.018
Positive RLNs, (+) vs. (−)	2.85	0.86–9.50	.088
Positive CLNs, (+) vs. (−)	2.53	0.80–8.01	.113
LN necrosis, (+) vs. (−)	1.22	0.59–2.52	.594
LNs with ES, (+) vs. (−)	0.57	0.27–1.19	.131
D_LN-IIa_ ≥ 20 mm or level IIa LNs with ES, (+) vs. (−)	2.21	1.10–4.46	.026
MAD of LNs ≥30 mm, (+) vs.(−)	1.51	0.70–3.25	.293
Positive bilateral CLNs, (+) vs.(−)	1.95	0.97–3.92	.061
Positive CLNs at supraclavicular fossa, (+) vs. (−)	2.04	0.87–4.82	.103

Abbreviations: *LNs*, lymph nodes; *HR*, hazard ratio; *95 % CI*, 95 % confidence interval; *RLNs*, retropharyngeal lymph nodes; *CLNs*, cervical lymph nodes; *D*_*LN-IIa*_, greatest dimension of level IIa lymph nodes; *MAD*, maximal axial diameter; *ES*, extra-capsular spread

*P-values were calculated using a binary logistic regression model

The percentage of positive level Ib LNs at diagnosis in patients with and without a DLN-IIa ≥ 20 mm or level IIa LNs with ES were 6.9 % vs. 1.7 % (*P* <.001); with and without oropharynx involvement, 7.8 % vs. 2.3 % (*P* = .001); and with and without positive bilateral CLNs, 6.7 % vs. 1.8 % (*P* <.001), respectively.

### Regional control at level Ib

Three patients experienced recurrence at level Ib, including two in-field recurrences (inside CTV2) and one out-of-field recurrence (outside CTV2). Table 3 shows the features of the three patients who suffered regional recurrence at level Ib; all three patients had a DLN-IIa ≥ 20 mm and/or level IIa LNs with ES, oropharynx involvement and/or positive bilateral CLNs at diagnosis. Therefore, the 904 patients without a DLN-IIa ≥ 20 mm level IIa LNs with ES, oropharynx involvement or positive bilateral CLNs at diagnosis were classified as patients at a low risk of metastasis to the level Ib LNs (low risk patients).

**Table 3:** Features of the three patients with recurrence at the level Ib LNs after intensity-modulated radiotherapy

		Case 1	Case 2	Case 3
Tumor involvement				
	Staging	T4N3a	T3N2	T4N3b
	Positive bilateral CLNs	Yes	Yes	Yes
	D_LN-IIa_ ≥20 mm or level IIa lymph nodes with ES	None	Right	Right
	Oropharynx involvement	Left	None	None
Irradiation of neck level Ib		Bilateral	Right	Right
Recurrence at neck level Ib				
	Laterality	Left	Right	Left
	Other regional recurrence	IA + IIb + IV + Vb	IIa + IIb + III	Ib
	Concomitant failure	Axillary LNs	-	Paranasophrynx+skull base
	Time to recurrence	12 months	12 months	23 months
	Salvage treatment	Chemo	Chemo + surgery	Chemo + RT
	Treatment response	PD	PD	PR
	Sequential failure	Death due to multiple metastasis	Axillary and mediastinal LNs	Death due to intractable epistaxis

Abbreviations: *LNs*, lymph nodes. *D*_*LN-IIa*_, greatest dimension of level IIa lymph nodes; *ES*, extra-capsular spread; chemo, chemotherapy; *RT*, radiotherapy; *PD*, progressive disease; *PR*, partial response

### Clinical characteristics of low risk patients

Table 3 shows the clinical characteristics of the 904 patients at low risk: 79.7 % (722/904) received level Ib-sparing IMRT and 20.1 % (182/904) received level Ib-covering IMRT. Significantly higher numbers of younger patients and patients with advanced N disease received level Ib-covering IMRT, and a significantly higher number of patients treated with level Ib-covering IMRT received chemotherapy (Table 4).

**Table 4:** Clinical features at diagnosis for low risk patients who received level Ib-sparing and -covering IMRT

Variable	Irradiation of level Ib, *N* (%)		*P* ^*^
	(−), *n* = 722	(+), *n* = 182	
Sex			
Male	536 (74.2)	122 (67.0)	.051
Female	186 (25.8)	60 (33.0)	
Age			
<50 years	447 (66.1)	139 (76.4)	.008
≧50 years	245 (33.9)	43 (23.6)	
T classification			
T1	157 (21.7)	36 (19.8)	.208
T2	108 (15.0)	38 (20.9)	
T3	332 (46.0)	83 (45.6)	
T4	125 (17.3)	25 (13.7)	
N classification			
N0	206 (28.5)	21 (11.5)	<.001
N1	493 (68.7)	137 (75.3)	
N3	20 (2.8)	24 (13.2)	
Positive RLNs			
(−)	256 (35.5)	45 (24.7)	.006
(+)	466 (64.5)	137 (75.3)	
Positive CLNs			
(−)	479 (66.3)	60 (33.0)	<.001
(+)	243 (33.7)	122 (67.0)	
Positive CLNs at supraclavicular fossa			
(−)	710 (98.3)	168 (92.3)	<.001
(+)	12 (1.7)	14 (7.7)	
Chemotherapy			
(−)	147 (20.4)	15 (8.2)	<.001
(+)	575 (79.6)	167 (20.1)	

Abbreviations: *IMRT*, intensity-modulated radiotherapy; *RLNs*, retropharyngeal lymph nodes; *CLNs*, cervical lymph nodes; *ES*, extra-capsular spread

* *P*-values were calculated using unadjusted chi-square test (or Fisher’s exact test, if indicated)

**Table 5:** Multivariate analyses of prognostic factors in low risk patients (*n* = 904)

Variable	LR-FFS		D-FFS		FFS		OS	
	HR (95 % CI)	*P* ^*^	HR (95 % CI)	*P* ^*^	HR (95 % CI)	*P* ^*^	HR (95 % CI)	*P* ^*^
Sex, female vs. male	0.68 (0.34–1.38)	.290	0.82 (0.46–1.42)	.459	0.82 (0.52–1.29)	.384	0.77 (0.37–1.63)	.499
Age, ≥50 vs. <50 years	1.27 (0.69–2.32)	.445	1.44 (0.87–2.37)	.155	1.60 (1.08–2.37)	.020	2.44 (1.29–4.60)	.006
T classification	1.51 (1.11–2.07)	.009	1.32 (1.03–1.70)	.029	1.33 (1.08–1.64)	.007	1.60 (1.12–2.28)	.009
Positive RLNs, (+) vs. (−)	1.70 (0.77–3.73)	.185	1.43 (0.76–2.70)	.266	1.55 (0.94–2.58)	.089	1.17 (0.53–2.57)	.694
Positive CLNs, (+) vs. (−)	2.16 (1.20–3.89)	.010	2.35 (1.40–3.96)	.001	2.01 (1.34–3.04)	.001	2.76 (1.44–5.32)	.002
Positive CLNs at SCF, (+) vs. (−)	1.16 (0.27–5.04)	.846	3.00 (1.24–7.18)	.014	2.12 (0.96–4.71)	.064	2.69 (0.79–9.12)	.113
Chemotherapy, (+) vs. (−)	1.14 (0.38–3.41)	.816	1.18 (0.48–2.91)	.719	0.89 (0.46–1.71)	.717	0.52 (0.20–1.34)	.174
Irradiation of level Ib, (+) vs. (−)	1.68 (0.88–3.19)	.114	1.43 (0.82–2.49)	.207	1.31 (0.83–2.05)	.247	0.88 (0.39–1.95)	.744

Abbreviations: *LR-FFS*, locoregional failure-free survival; *D-FFS*, distant failure-free survival; *FFS*, failure-free survival; *OS*, overall survival; *HR*, hazard ratio; *95 % CI*, 95 % confidence interval; *RLNs*, retropharyngeal lymph nodes; *CLNs*, cervical lymph nodes; *D*_*LN-IIa*_, greatest dimension of level IIa lymph nodes; *LNs*, lymph nodes; ES, extra-capsular spread

^*^P-values were calculated using an adjusted Cox proportional-hazards model

### Patterns of failure for low risk patients

Median follow-up time for the low risk patients was 38.7 months (range, 1.3–57.8 months); 63.6 % (631/904) were followed up for ≥ 3 years. In total, 11.4 % (113/904) of the low risk patients developed treatment failure: distant metastasis was the most common pattern of failure (65/ 904 patients; 7.2 %); 3.3 % (30/904) experienced local failure; 2.1 % (19/904) experienced regional recurrence, including 1/23 (5.3 %) at level Ia, 0/23 at level Ib (0 %), 11/19 at level II (57.9 %), 4/19 at level III (21.0 %), 2/19 at level IV (10.5 %), 1/19 at level V (10.5 %). Twelve of the 904 low risk patients (1.3 %) developed both distant failure and locoregional recurrence. At last follow-up, 39 deaths had been recorded in the 904 low risk patients (4.3 %), with the majority (31/39, 88.6 %) attributed to NPC.

### Survival outcomes of low risk patients

The estimated 3-year LR-FFS, D-FFS, FFS, and OS rates for low risk patients were 95.5 %, 92.8 %, 89.2 %, and 96.4 %, respectively. Significant differences were observed in the estimated 3-year survival rates between low risk patients who received level Ib-sparing IMRT and level Ib-covering IMRT (LR-FFS: 96.2 % vs. 92.0 % [HR 1.92; 95 % CI 1.04–3.56; *P* = .013]; D-FFS: 93.9 % vs. 88.2 % [HR 1.92; 95 % CI 1.14–3.23; *P* = .012]; FFS: 90.6 % vs. 84.1 % [HR 1.64; 95 % CI 1.08–2.51; *P* = .022]; OS: 96.5 % vs. 96.1 % [HR 1.18; 95 % CI 0.56–2.49; *P* = .662], respectively, Table 5). However, in multivariable analyses, irradiation of level Ib was not an independent risk factor for LR-FFS, D-FFS, FFS or OS (Table 5).

### Xerostomia in low risk patients

In total, 50.7 % (463/913) of the low risk patients experienced subjective xerostomia at 12 months after radiotherapy, which was predominately mild (grade I-II, 98.7 %). No significant difference was observed in the frequency of grade ≥ 2 subjective xerostomia at 12 months after radiotherapy among low risk patients who received level Ib-sparing, unilateral level Ib-covering or bilateral level Ib-covering IMRT (10.1 % vs. 14.0 % vs. 18.0 %, *P =* .056).

## Discussion

This is the largest-sample observational cohort study to assess clinical predictors of metastasis to the level Ib LNs in patients with NPC at diagnosis and furthermore, first to compare disease control and xerostomia after level Ib-sparing IMRT and level Ib-covering IMRT. We found that a DLN-IIa ≥ 20 mm and/or level IIa LNs with ES, oropharynx involvement and positive bilateral CLNs were independently significantly associated with metastasis to the level Ib LNs at diagnosis. These pretreatment factors effectively identify patients at low risk of recurrence at the level Ib LNs. For low risk patients, irradiation of level Ib was not an independent risk factor for LR-FFS, D-FFS, FFS or OS.

The incidence of level Ib LN metastasis in this study was only 2.8 %, which is similar to previous studies [11, 13–15]. Based on previous research [26–28], we hypothesized primary tumor invasion and nodal disease may be related to metastasis to the level Ib LNs. In our analyses, a DLN-IIa ≥ 20 mm and/or level IIa LNs with ES, oropharynx involvement and positive bilateral CLNs were independently significantly associated with level Ib LN involvement at diagnosis, in accordance with previous studies [26–28]. The level Ib LNs receive efferent lymphatic drainage from the submental LNs, medial canthus, lower nasal cavity, hard and soft palates, maxillary and mandibular alveolar ridges, cheek, upper and lower lips, and most of the anterior tongue [12, 29]. The level Ib LNs are at risk of developing metastases from cancers of the oral cavity, anterior nasal cavity, soft tissue structures of the middle face, and SMGs. Therefore, we concluded that level Ib is not a regular region of direct drainage for the primary tumor in NPC. We speculate level Ib involvement may result from retrograde tumor spread after blockage of the normal routes of lymphatic drainage (for example, massive level IIa LNs or bilateral positive CLNs), or metastasis from tumors involving anatomical sites that drain to level Ib (for example, the oropharynx, which is adjacent to the soft palate). However, similarly to previous studies [26–28], nasal cavity involvement did not correlate with metastasis to level Ib in this study. This may be explained by the fact that the above-mentioned studies did not include involvement of the anterior nasal cavity as a variable for analysis. Nasal cavity involvement did not exceed the posterior third in axial plane on MRI scans in most cases in this study, and only the anterior third of the nasal cavity drains to level Ib [12].

Though various protocols of level Ib delineation and dose definitions for IMRT have been reported at different treatment centers over the years [1, 11, 16–21, 30], there is little evidence to address the association between elective irradiation and disease control at level Ib. Chen and colleagues [22] investigated 120 patients with NPC and negative level Ib LNs at diagnosis who received level Ib-sparing IMRT and observed no regional recurrence at level Ib, and regional LN recurrence alone was rare. They concluded that level Ib-sparing IMRT is feasible in patients with negative level Ib LNs [22]. Yi et al. [27] developed a risk score model for metastasis to the level Ib LNs and found that level Ib-sparing irradiation was an independent risk factor for locoregional recurrence in 190 high risk patients (involvement of level II/III/IV LNs, carotid sheath involvement and the maximal axial diameter [MAD] of the CLNs ≥ 20 mm). However, level Ib-sparing irradiation did not affect locoregional recurrence in the 137 low risk patients in the same study. However, it should be noted that all of the 327 patients received three-dimensional conventional radiation therapy (3D-CRT), which is inferior to IMRT in terms of OAR protection [31, 32], and data on xerostomia was not available to confirm the advantage of level Ib-sparing irradiation [27].

Interestingly, all the three cases of level Ib LN recurrences in this study occurred in patients with a DLN-IIa ≥ 20 mm, level IIa LNs with ES, oropharynx involvement and/or positive bilateral CLNs at diagnosis. According to our previous analysis, though 79 % of low risk patients were treated with level Ib-sparing IMRT, none of these patients experienced recurrence at level Ib. Our multivariable analyses also showed that irradiation of level Ib was not an independent risk factor for LR-FFS, D-FFS, FFS or OS. Omitting irradiation of level Ib did not significantly jeopardize disease control at level Ib nor compromise locoregional control, distant control or OS in low risk patients in this study. Therefore, we conclude that level Ib-sparing IMRT should be safe in patients without a DLN-IIa ≥ 20 mm, level IIa LNs with ES, oropharynx involvement or positive bilateral CLNs. Our results are in accordance with previous studies [22, 27] and provide further meaningful evidence for elective sparing of level Ib in the IMRT era.

Previous studies have reported level Ib-sparing IMRT reduces xerostomia in patients with head and neck cancer [6–8, 10]. However, this study did not observe a significant difference in the frequency of grade ≥ 2 subjective xerostomia at 12 months after IMRT between patients who received level Ib-sparing, unilateral level Ib-covering or bilateral level Ib-covering IMRT. The main reason for this result is that the dose constrains for the SMGs were not included in the treatment planning protocol of our centre. Even when the SMGs were excluded from the CTV2, the 40 Gy isodose line still exceeded the anterior two-thirds of the SMGs in this series, while previous studies reported that the SMG salivary flow rate depends on the mean dose to the SMGs up to a threshold of 39 Gy, with recovery over time [8]. Investigations of SMG-sparing IMRT also found it feasible to substantially reduce the dose to the SMG to below a threshold of 39 Gy without target underdosing [8]. Therefore, we believe that proper dose constrains for the SMGs should be studied in the future for level Ib-sparing IMRT in certain cohorts of patients with NPC.

This is the largest sample size study to investigate the feasibility of elective level Ib-sparing IMRT. However, this study inevitably bears the inherent limitations of its retrospective nature. Firstly, the identification of low risk patients who may not need irradiation to level Ib was not based on pathologic evidence but assessment of pretreatment MRI scans. For example, ES was diagnosed on the basis of radiographic findings, which is a common and difficult problem for NPC research due to the lack of pathologic confirmation of LN metastases in patients with NPC. Secondly, irradiation of level Ib was not randomly assigned but decided by the individual physicians for each patient, based on their recognition of the delineation protocols from reports of different centers. Bias towards more patients with advanced N disease receiving level Ib-covering IMRT was inevitable. Thirdly, delineation of the SMGs was not described in the treatment planning protocol of our centre; therefore, further analyses of the relationship between the degree of xerostomia and dose to the SMGs was not possible for this cohort. Further investigations based on more specific criteria for dose constraints for the SMGs are warranted to confirm the benefit of elective level Ib irradiation.

## Conclusion

Level Ib-sparing IMRT should be safe and feasible for patients without a DLN-IIa ≥ 20 mm and/or level IIa LNs with ES, positive bilateral CLNs or oropharynx involvement at diagnosis. Further investigations based on specific criteria for dose constraints for the SMGs are warranted to confirm the benefit of elective level Ib irradiation.

## Abbreviations

NPC: Nasopharyngeal carcinoma
IMRT: Intensity-modulated radiation therapy
LN: Lymph node
CLN: Cervical lymph nodes
RLN: Retropharyngeal lymph node
DLN-IIa: Greatest dimension of level IIa LNs
ES: Extracapsular spread
PGs: Parotid glands
SMGs: Submandibular glands
GTV: Gross tumor volume
CTV: Clinical target volumes
PTV: Planning target volume
